# Coupling coordination degree and influencing factors of forestry modernization and high quality economic development: an empirical study from provincial panel in China

**DOI:** 10.3389/fpubh.2024.1436292

**Published:** 2024-10-10

**Authors:** Chao Zhou, Shenwei Wan, Jia Liu, Ye Ma, Hongling Zheng, Song Chen

**Affiliations:** ^1^Research Center of the Economic and Social Development of Henan East Provincial Joint, Shangqiu Normal University, Shangqiu, China; ^2^School of Agricultural Economics and Rural Development, Renmin University of China, Beijing, China; ^3^School of Economics and Management, Northeast Forestry University, Harbin, China; ^4^School of Finance and Business, Shanghai Normal University, Shanghai, China; ^5^School of Ocean, Tangshan Normal University, Tangshan, China; ^6^College of Marxism, Yunnan Agricultural University, Kunming, China

**Keywords:** forestry modernization, high quality economic development, coupling coordination degree, influencing factors, Tobit

## Abstract

**Introduction:**

Forestry modernization (FM)^1^ and High quality economic development (HED)^2^ are two major goals that must be achieved in the process of economic development from low to high, and they are closely related to each other in their respective internal development mechanisms.

**Methods:**

Based on the analysis of the coupling coordination mechanism between FM and HED, this paper empirically studies the coupling coordination degree and influencing factors of “forestry modernization and high quality economic development”^3^ (“FM-HED”) using panel data from 30 provinces and cities in China (except Xizang, Hong Kong, Macao and Taiwan) from 2012 to 2020. First, the entropy method and the coupling coordination degree model were used to analyze the temporal changes and spatial differentiation of the coupling coordination between FM and HED. Second, the Tobit model is used to find out the factors affecting the coupling coordination degree of FM and HED.

**Results:**

The following conclusions are obtained: (1) The coupling coordination degree of the “FM-HED” system increased rapidly in the early stage and slowly increased in the later period from 2012 to 2020. (2) The level of FM and HED in China in 2012 was obviously unbalanced and insufficient, the coupling coordination degree between the eastern and western provinces and cities was improved in 2015, and the imbalance between regions was alleviated, and the difference in the coupling coordination degree between coastal provinces and cities and inland provinces and cities in 2020 was prominent, and the coastal areas were significantly better than the inland areas. (3) From the national level, the intensity of R&D investment and the burden of the older adult population have a significant effect on the coupling and coordinated development of the two systems. From the perspective of the four regions, the role of R&D investment intensity is reflected in Northeast China and Central China. The role of labor force level is reflected in East China, Northeast China and Northwest China. The role of industrial agglomeration is reflected in Northeast China. The role of the burden of the older adult population is reflected in Northeast China. The role of government intervention is reflected in East China and Northeast China. Accordingly, this paper puts forward corresponding policy suggestions to better promote the coupling and coordinated development of FM and HED.

## 1 Introduction

Forestry is an important resource-based industry and environmental protection industry, with high economic and ecological value, and plays an important role in the green, low-carbon and sustainable development of the economy and society (1). Although China’s forestry development has made great achievements that have attracted worldwide attention, there are still some problems that cannot be ignored in the traditional forestry production mode, such as high consumption, low efficiency, sharp reduction of natural forest resources, single afforestation tree species, unreasonable forestry industry structure and low forestry economic efficiency (2). In order to solve this dilemma, China’s first Central Forestry Work Conference clearly pointed out: “Forestry has an important position in the implementation of the strategy of sustainable development (3), a primary position in ecological construction, a basic position in the large-scale development of the western region, and a special position in responding to climate change” (4). As China has incorporated “Beautiful China” into the goal of becoming a modern and powerful country, and “Ecological Civilization” into the overall layout of economic, political, cultural and social construction, FM has entered the public eye as the main position of building “Beautiful China” and “Ecological Civilization” (5).

Promoting FM is not only an inevitable requirement for improving the quality of forestry development, but also a potential for achieving HED (6). On the one hand, FM promotes HED. HED is inseparable from the five new development concepts of “innovative, coordinated, green, open and shared” (7). With the transformation of China’s economic development from emphasizing speed to emphasizing quality, the high quality development of the forestry industry has continuously promoted the development of the modern economy in the direction of innovative, coordinated and green^4^, and the import and export trade of forest products has also contributed to the open and shared of China’s economy (8). On the other hand, HED feeds back FM. FM is a long-term accumulation and development process, these changes rely on the organic combination of resources, environment, science and technology, system, law and many other modern factors, a solid economic foundation is the fundamental guarantee to achieve forestry from quantitative change to qualitative change.^5^

Based on the above analysis, studying the relationship between FM and HED is not only beneficial for China to promote the transformation and upgrading of the forestry industry while ensuring the growth of forest resources and forest ecological security, thereby stimulating the overall HED of the regional economy, but also beneficial for developing countries to gradually promote the process of FM under the premise of abundant forest resource endowment, thereby driving regional economic development and solving the problem of “forest resource curse” with solutions from China. The coupling coordination degree between FM and HED describes the degree of mutual promotion and coordinated development between these two systems. This indicator puts two research objects into one research framework, highlighting the interactions between closely related parts of the national economic development. Specifically, the low coupling coordination between FM and HED indicates that there is no mutual promotion between them, and each part is in its own development state, indicating that economic development is not coordinated. The high coupling coordination between FM and HED indicates that they have achieved mutual promotion, and the development of various parts of the national economy has formed a mutually promoting effect, indicating that the economic development is relatively coordinated. Sorting out the relationship between the two and identifying the factors that affect their coordinated development not only expands the relevant theories of their combined research, clarifies the relationship between FM and HED, but also has important practical significance for developing countries to coordinate the relationship between FM and HED in the future, and achieve the sustainable goal of green development.

## 2 Literature review

The research of domestic and foreign scholars on FM mainly focuses on the following four aspects: the construction of FM measurement system (9), the dynamic evolution process and characteristics of China’s FM (10), the exploration of FM goals and paths in state owned forest areas (11), and the research on the promotion of FM by the development of informatization and modern technology (12). The research of domestic and foreign scholars on HED mainly focuses on digital economy, green finance, scientific and technological innovation, regional economy, industrial synergy and countermeasure research (13). FM is an important link related to HED, and HED is an indispensable guarantee and assistance for FM, and a coupling system has been formed between the two. Through the study of the coupling coordination degree between systems, some scholars have found that the spatial correlation between HED and other systems is not strong, and the spatial spillover effect is weak (14). At the provincial and regional levels, there is a great deal of spatial heterogeneity in the index and coupling coordination of the FM and HED systems. Some scholars pointed out that the realization of the goal of “carbon neutrality and carbon peaking” and HED are the two main tasks in the context of Chinese style modernization, and they are also the inevitable choice to promote the transformation of FM (15). Correspondingly, FM is also an important support for the realization of these two major tasks. There is a systematic coupling relationship between FM and HED (16).

Compared to existing research, our marginal contribution may be as follows: First, the concepts of FM and HED are relatively vague, and existing studies have only explored their conceptual definitions and theoretical connotations, without constructing a rigorous indicator system (17). Our research builds indicator systems for FM and HED from multiple dimensions based on existing theories; Second, most existing research only focuses on the FM or HED, without capturing the correlation between the two systems (18). There is also no research that measures the coupling coordination degree of the two systems. We mainly use the entropy method to weight and calculate the comprehensive index of the sub indicators of the two systems based on panel data from 30 provinces and cities in China from 2012 to 2020. We calculate the coupling coordination degree between the two systems and analyze its spatiotemporal evolution trend, which has certain guiding significance for breaking the “forest resource curse” and achieving green economic development; Third, most current research on forestry ecosystems and economic development systems only uses case analysis methods in terms of methodology, and has not been able to test both through data (19); In combination with the limitations of the explained variable (the characteristics of values between 0 and 1), we set the panel Tobit model to dig out many influencing factors that affect the stable growth of the coupling coordination degree of FM and HED, and pay attention to the heterogeneity between regions, which provides a theoretical basis for promoting the realization of the value of forest ecological products according to local conditions, boosting Chinese path to modernization, and providing experience for developing countries to achieve sustainable development goals.

Based on this, the research structure of this paper is as follows: The first part, introduction. It is used to introduce the concept analysis and background of FM and HED. The second part, literature review. This paper summarizes the research on FM and HED, and summarizes the possible marginal contributions of this paper. The third part is mechanism analysis. Referring to the existing research results, the principle and influence path of coupling and coordination between FM and HED were analyzed. The fourth part is empirical analysis. This part covers four points: indicator selection, data sources, model selection and result analysis. Among them, the entropy method and the coupling coordination degree model are used to analyze the temporal changes and spatial differentiation of the coupling coordination between FM and HED, and the Tobit model is used to explore the influencing factors of the coupling coordination degree between FM and HED. The fifth part, conclusions and policy suggestions.

## 3 Mechanism analysis of coupling coordination between FM and HED

### 3.1 Relationship between FM and HED

FM and HED are two independent, open and interdependent complex systems, and many factors constituting the system of “FM-HED” promote each other and develop together, so it is of great significance to analyze the interaction mechanism between the two to accelerate FM, improve the level of HED and improve the efficiency of system interaction.

FM contributes to HED. Forestry is an important part of the national economy, and the economic benefits created by forestry directly affect the quantity and quality of the national economy, and this influence mainly comes from the following five aspects.

First, FM is one of the main contents of Chinese modernization, which promotes economic innovation and development (20). The investment in forestry science and technology and the promotion of forest and grassland science and technology are of great significance to the modernization of forestry, and the investment of scientific and technological elements in forestry will significantly promote the improvement of the innovation level of forest areas, thus becoming the core driving force of economic development.

Second, FM serves rural revitalization and promotes coordinated economic development. Most rural areas often have a “forest curse” of abundant forest resources but low level of economic development, and FM is the key to solving this problem (21).

Third, forest carbon sequestration is an important driving force for the development of a low-carbon economy and promotes the green development of the economy. In the context of climate change, energy security and international competition, the low-carbon economy is expected to be high, and the continuous improvement of the forest carbon sink market is the main content of FM transformation, which can maximize the reduction of enterprises to high-carbon energy consumption, and is the key influencing factor for the sustainable development of low-carbon economy (22).

Fourth, FM will help the construction of the Belt and Road Initiative and promote the opening up and development of the economy. In recent years, China has established multi-faceted forestry science and technology cooperation with countries along the “Belt and Road,” which is an important way to further promote the opening up and development of China’s economy.

Fifth, the realization of the value of forest products creates direct economic benefits for rural residents and promotes the development of economic sharing. Most of China’s vast forests are located in mountainous areas and rural areas, and the forest resources of forest land continue to grow, and the forest wealth continues to increase, and the level of forestry development is essentially the concrete embodiment of the economic value of forestry. FM can not only improve the efficiency of forestry production, but also directly affect the quality of economic development in rural areas and promote the realization of common prosperity (23).

HED has forced the modernization of forestry. With the improvement of the level of economic and social development and the successive proposals of the goal of “carbon peaking and carbon neutrality,” Chinese people have higher and higher requirements for a better ecological environment, and inevitably have higher expectations for forestry development. Specifically, this kind of reversal can be reflected in the following five aspects.

First, HED requires social and economic needs to be green, forcing forestry ecology. The continuous improvement of the quality of economic development has also put forward higher requirements for forest ecological environment and ecosystem services, and promoted the effective development of more modern afforestation projects, thus effectively increasing the quantity and quality of forests.

Second, HED requires the realization of the value of forest ecological products, forcing forestry industrialization. One of the important contents of HED is the development of the digital economy, and the market channels for realizing the value of forest ecological products under the emerging forms of the digital economy have been continuously dredged, so as to promote the effective transformation of forest ecological products from “lucid waters and lush mountains” to “golden mountains and silver mountains,” thereby boosting the upgrading of the forestry industry structure and forming a new ecological forestry industry.

Third, HED requires the improvement of the level of forestry socialized services, which forces forestry socialization. In the context of comprehensively promoting rural revitalization, forestry revitalization is also an important part of ecological revitalization in rural revitalization. With the gradual narrowing of the income gap between urban and rural areas and the deepening of urbanization, the equalization of social services of rural forestry and urban forestry needs to be supported by FM, and only modern forestry social services can meet the expectations of urban and rural residents for the development of forestry ecological resources and forestry livelihoods, so as to meet the requirements of equalization of urban and rural forestry ecological welfare.

Fourth, HED needs the support of the forestry tourism industry, forcing forestry tourism. The traditional forestry industry has been unable to realize the inevitable requirements of FM, forestry tourism is mainly reflected in the development of forestry services such as understory economy, forest health care, forest tourism and forest research, which needs to rely on the reform of collective forest rights, cultivate new forestry management entities such as large forestry households, family forest farms, forestry cooperatives, revitalize forest land management rights, revitalize financing channels, and drive forestry tourism to support HED. Through forestry tourism, the path to realize the value of forest ecological products will be opened up, and the high quality development of forestry economy will be promoted.

Fifth, the improvement of the quality of economic development can also force forestry enterprises to eliminate backward forestry machinery and production capacity equipment, and force forestry science and technology. A forestry power is the fundamental requirement for HED, digital empowerment is an important starting point for building a forestry power, and forestry science and technology is the only way to improve digital technology. Therefore, it is necessary to realize the innovation drive of the forestry industry, improve the degree of forestry marketization, and build smart forestry and digital forestry with high and new technologies, so as to promote the development of forestry science and technology and lay a good scientific and technological foundation for HED.

### 3.2 Main aspects affecting the coordinated development of FM and HED

Among the factors affecting the coordinated development of FM and HED, a review of existing research reveals the following main aspects (24, 25):

First, the intensity of research and development investment has a positive impact on the coupling degree between FM and HED. The increase in R&D investment intensity can promote technological innovation, optimize resource allocation, and drive industrial upgrading, thereby enhancing the coordinated development and overall efficiency within the economic system.

Second, the number of employed people can reflect the activity level of the labor market, and the level of labor employment can reflect the effective allocation of resources and stable economic development. This stability helps maintain the stability of the coupling relationship between FM and HED.

Third, the increase in the degree of industrial agglomeration helps to enhance the interaction and coordination among various elements within the economic system, forming economies of scale and synergies, which in turn helps to improve the coupling level between FM and HED.

Fourth, the higher the proportion of people participating in pension insurance in the total population of the region, the higher the burden on the older adult population. As the proportion of older adult population increases, social security expenditures will significantly increase, which will lead to increased financial pressure on the government, affecting the stability and development of the social security system, and may also affect the coupling degree between FM and HED.

Fifth, the government can guide resources toward key areas and weak links through fiscal expenditures, promote industrial upgrading and social development, optimize resource allocation, help improve the operational efficiency of the economic system, and enhance the coupling degree between FM and HED.

**Figure 1:**
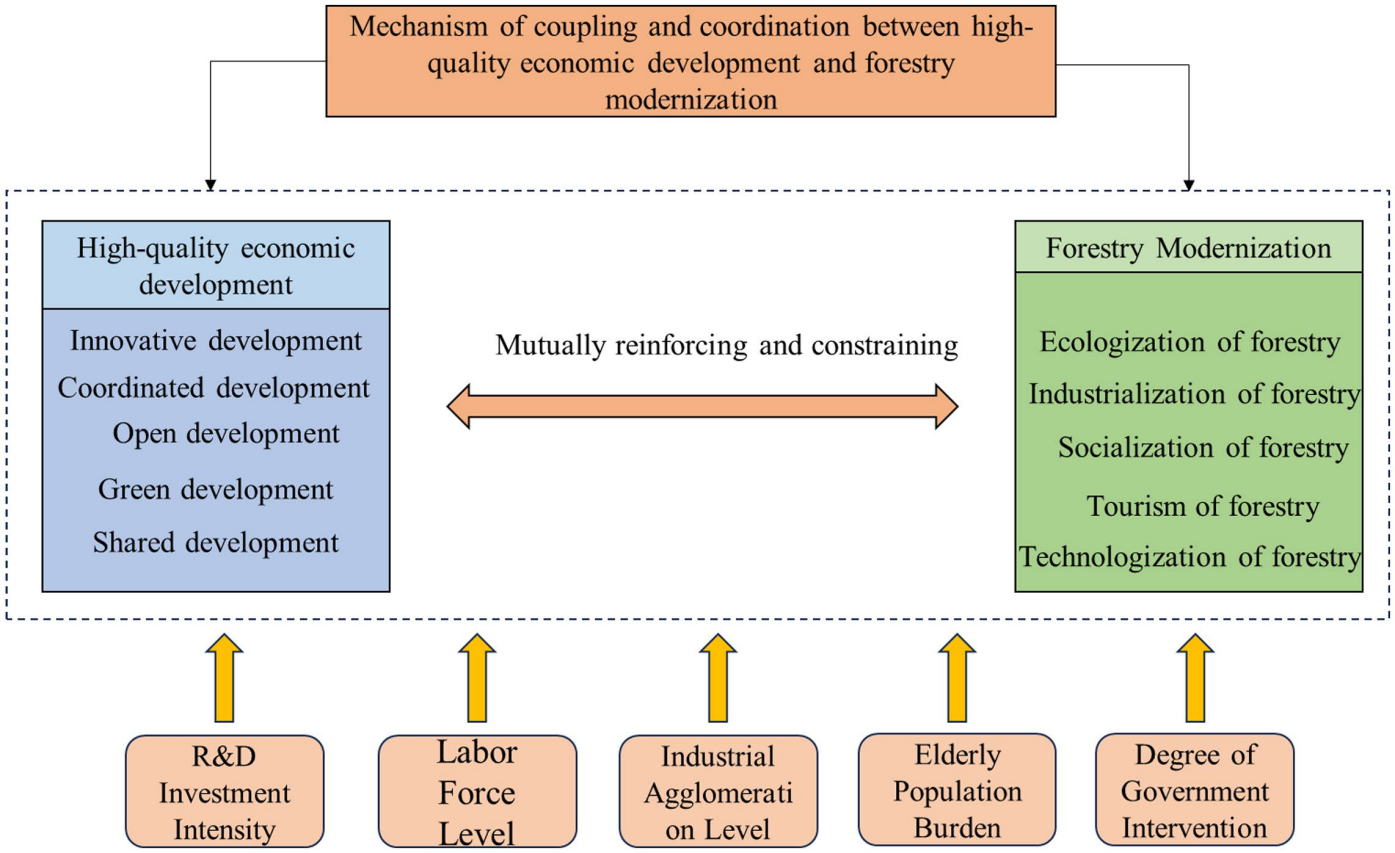
Mechanism of coupling coordination between HED and FM.

The specific theoretical logic diagram is shown in Figure 1.

## 4 Empirical analysis of the coupling coordination of FM and HED

In this paper, 30 provinces and cities in China (except Xizang, Hong Kong, Macao, and Taiwan) from 2012 to 2020 were taken as the research objects, and the evaluation index system of coupling and coordinated development of “FM-HED” was constructed, and then the entropy method and coupling coordination model were used to analyze the temporal changes and spatial differentiation of the coupling coordination between FM and HED, and the Tobit model was used to find the factors affecting the coupling coordination degree of FM and HED.

### 4.1 Indicator selection

Constructing a scientific and complete evaluation index system for coupling and coordinated development is the basis and premise for judging the level of coupling coordination degree of “FM-HED.” However, at present, the relevant evaluation indicators of FM and HED have their own emphasis, and a set of recognized evaluation index systems has not yet been formed. Therefore, in order to ensure that the selected indicators can comprehensively and objectively reflect the coupling coordination relationship between FM and HED. First, this paper analyzes the FM system layer and the HED system layer based on the principles of scientificity, comprehensiveness and operability, and divides them into five first-level indicators. Second, the widely used high-frequency indicators were screened and referenced with reference to relevant research results to form a detailed secondary index framework. Third, according to the research direction and framework of this paper, the index system of this paper is formulated.

FM should be comprehensive. The Decision of the Central Committee of the Communist Party of China and the State Council on Accelerating the Development of Forestry (hereinafter referred to as The Decision) pointed out that the FM will take the path of civilized development with production development, affluent life and good ecology as the key, so as to achieve the coordination of social economy with population, resources and environment. The Decision focuses on the sustainable management of forestry, key projects of ecological construction, the long-term livelihood of farmers and ecological migrants who have returned farmland to farmland, the reform of the forestry system and the upgrading of the forestry industry structure, and the revitalization of forestry through science and education. Based on The Decision, and referring to the relevant research results of Zambon et al. (26), to measure the development level of FM, this paper subdivides the FM system into five first-level indicators: forestry ecology, forestry industrialization, forestry socialization, forestry tourism and forestry science and technology. The selection of secondary indicators fully reflects the ecological, production and living functions of forestry, and the weight of forestry industry development level is larger, exceeding 0.1, as shown in Table 1. The characteristics of HED are embodied in the five new development concepts of “innovative, coordinated, green, open and shared “, which are the most basic reference standards widely adopted by the academic community (27). From the above five aspects, the corresponding indicators were selected to evaluate the level of HED, and a first-level evaluation index system was formed. Most of the secondary indicators at the system level of HED are positive indicators, referring to the index system of Cheng et al. (28), to measure the level of HED, the GDP growth rate, demand structure, energy consumption elasticity coefficient, foreign trade dependence, and the proportion of labor compensation are selected as the secondary indicators to reflect the economic development status, as shown in Table 2.

**Table 1:** Evaluation index system for coordinated development of the coupling of “FM.”

System layer	Primary indicator	Secondary indicator	Indicator definition and unit	Weightage
Forestry Modernization(FM)	Forestry Ecologization	Forest Ecological Level (+)	Forest Coverage Rate (%)	0.0195
		Afforestation Ecological Level (+)	Afforestation Total Area (Thousand Hectares)	0.0276
		Forest Ecological Level (+)	Forest Stock Volume (Billion Cubic Meters)	0.0507
		Protected Area Forestry Ecological Level (+)	Protected Area (Ten Thousand Hectares)	0.0755
		Forest Land Ecological Level (+)	Forestry Land Area (Ten Thousand Hectares)	0.0323
	Forestry Industrialization	Forest Industrialization Level (+)	Forestry Added Value (Billions of Yuan)	0.0327
		Timber Industrialization Level (+)	Timber Production (Ten Thousand Cubic Meters)	0.0625
		Forestry Industrial Investment Intensity (+)	Forestry Investment (Thousands of Yuan)	0.0396
		Forestry Industrial Development Level (+)	Forestry Industrial Development Yearly Completed Investment (Thousands of Yuan)	0.1007
	Forestry Socialization	Rural Resident Household Forestry Social Service Level (+)	Rural Resident Household Fixed Assets Original Value for Forestry Production (Yuan/Household)	0.0798
		Park Forestry Socialization Degree (+)	Park Green Area (Ten Thousand Hectares)	0.0276
		Forest Ranger Socialization Level (+)	Full-Time Forest Ranger Count (Individuals)	0.0451
	Forestry Tourism	Forestry Tourism Intensity (+)	Forestry Tourism Visitors (Number of People)	0.0453
		Forestry Tourism Level (+)	Forestry Tourism Income (Ten Thousand Yuan)	0.0624
		Forestry Tourism Degree (+)	*Per Capita* Forestry Tourism Expenditure (Yuan)	0.0191
		Forestry Tourism Effectiveness (+)	Other Industries’ Output Value Directly Driven by Forestry Tourism (Ten Thousand Yuan)	0.0687
	Forestry Technologization	Rural Resident Household Forestry Technology Service Level (+)	Rural Resident Household Machinery Original Value for Forestry (Yuan/Person)	0.0592
		Forestry and Grass Technology Promotion Intensity (+)	Number of Forestry Economic Cooperatives Guided or Supported by Forestry and Grass Technology Promotion (Units)	0.0520
		Forestry and Grass Technology Promotion Effect (+)	Number of Households Driven by Forestry and Grass Technology Promotion (Households)	0.0463
		Forestry and Grass Technology Promotion Degree (+)	This Year’s Forestry and Grass Technology Promotion Area (Thousand Hectares)	0.0534

**Table 2:** Evaluation index system for coordinated development of the coupling of “HED.”

System layer	Primary indicator	Secondary indicator	Indicator definition and unit	Weightage
High-quality Economic Development(HED)	Economic Innovation Development	GDP Growth Rate (+)	Regional GDP growth rate (%)	0.0073
		R&D Investment Intensity (+)	R&D expenditure of industrial enterprises above designated size/GDP (absolute value)	0.0574
		Investment Efficiency (+)	Investment rate/regional GDP growth rate (absolute value)	0.0039
		Technological Transaction Activity (+)	Technology transaction volume/GDP (absolute value)	0.2006
		Demand Structure (+)	Total retail sales of social consumer goods/GDP (absolute value)	0.0153
	Economic Coordinated Development	Urban–Rural Structure (+)	Urbanization rate (%)	0.036
		Government Debt Burden (−)	Government debt balance/GDP (absolute value)	0.0833
		Industrial Structure (+)	Increase in the proportion of the tertiary industry’s output value to regional GDP (absolute value)	0.0455
		Energy Consumption Elasticity (−)	Energy consumption growth rate/GDP growth rate (absolute value)	0.0224
	Economic Green Development	Wastewater per Unit Output (−)	Wastewater discharge/GDP (absolute value)	0.0189
		Air Pollutants per Unit Output (−)	Sulfur dioxide emissions/GDP (absolute value)	0.1484
		Dependence on Foreign Trade (+)	Total import and export volume/GDP (absolute value)	0.1223
	Economic Openness Development	Foreign Investment Proportion (+)	Foreign investment total/GDP (absolute value)	0.1231
		Degree of Marketization (+)	Regional marketization index (absolute value)	0.0329
		Proportion of Worker Compensation (+)	Worker compensation/regional GDP (absolute value)	0.0235
	Economic Shared Development	Elasticity of Household Income Growth (+)	*Per capita* disposable income growth rate/regional GDP growth rate (absolute value)	0.0122
		Urban–Rural Consumption Gap (−)	Urban *per capita* consumption expenditure/Rural *per capita* consumption expenditure (absolute value)	0.0320
		Proportion of Livelihood-Focused Fiscal Expenditure (+)	Proportion of expenditure on housing security, medical care, local fiscal education, social security, and employment in local fiscal budget expenditure (%)	0.0150

### 4.2 Data sources

In this study, panel data from 30 provinces, autonomous regions and municipalities (excluding Xizang, Hong Kong, Macao and Taiwan) from 2012 to 2020 were selected from the China Urban Statistical Yearbook (2012–2020), China Urban Construction Statistical Yearbook (2012–2020), China Energy Statistical Yearbook (2012–2020), and China Forestry and Grassland Statistical Yearbook (2012–2020) and prefecture-level County Statistical Yearbooks (2012–2020), and the linear interpolation method of adjacent years was used to fill in the missing data.

### 4.3 Research methods

#### 4.3.1 Entropy method

This paper uses the entropy method to measure the comprehensive development index of China’s HED and FM, and its main calculation steps are as follows:

Step 1: Standardize the data to make the original data dimensionless, and refer to the following data processing methods for positive and negative indicators.

For positive indicators:


(1) $$\tilde{x_{ij}}=\frac{x_{ij}-\min\left\{x_{ij}\right\}}{\max\left\{x_{ij}\right\}-\min\left\{x_{ij}\right\}}$$

For negative indicators:


(2) $$\tilde{x_{ij}}=\frac{\max\left\{x_{ij}\right\}-x_{ij}}{\max\left\{x_{ij}\right\}-\min\left\{x_{ij}\right\}}$$

Equations 1 and 2, $\tilde{x_{ij}}$ refers to the *i*th sample of the *j*th indicator, *i* = 1, 2…, m; *j* = 1, 2, …, n; max {x*_ij_*} and min {x*_ij_*} refer to the maximum and minimum sample values for indicator *j*, respectively.

Step 2: By using $R_{ij}=\frac{x_{ij}}{\sum_{i=1}^{m}x_{ij}}$ to distort the indexed value, and then using $G_{ij}=1+\frac{\sum_{i=1}^{m}R_{ij}\times \ln R_{ij}}{\ln m}$ to calculate the importance of the *j*th indicator, that is, the coefficient of difference *Gj_._*

Step 3: Determine the weight of the selected indicator $\omega_{j}$ through $\omega_{j}=\frac{G_{ij}}{\sum_{j=1}^{a}G_{ij}}$ .

#### 4.3.2 Coupling coordination model

The so-called coupling relationship refers to a certain relationship between multiple (two or more) systems that are related and influenced by each other. The correlation and influence between the two major systems of HED (U1) and FM (U2) are defined as coupling coordination, and the degree of coordinated development of the two systems is defined as coupling coordination degree.

System Development Model:


(3) T=αU1+βU2

System Coordination Model:


(4) $$C=\frac{4U1\cdot U2}{{\left(U1+U2\right)}^{2}}$$

System Coupling Model:


(5) $$D=\sqrt{C\cdot T}$$

Equations 3–5, T represents the development degree of the comprehensive index of HED and FM, and U1 and U2 represent the comprehensive index results of HED and FM calculated by the entropy method, respectively. *α*、*β* are the weight coefficient to be determined, and α + β = 1. Considering that HED and FM are equally important in this study, the α = β = 0.5 was selected. C is the degree of coordination, which can judge the interrelationship between the two systems. D is the coupling degree of the two systems of HED and FM. At the same time, the coupling coordination degree is classified into different levels and classified into the corresponding coordination stages (Table 3).

**Table 3:** Classification of coupling coordination levels.

Coupling coordination	Coordination level	Coordination phase	Representative value
(0.0, 0.1]	Extreme dysregulation	Dysfunctional recession	1
(0.1, 0.2]	Severe dysregulation		2
(0.2, 0.3]	Moderate dysregulation		3
(0.3, 0.4]	Mild dysregulation		4
(0.4, 0.5]	On the verge of dysregulation	Transitional coordination category	5
(0.5, 0.6]	Barely coordination		6
(0.6, 0.7]	Primary coordination	Coordinated development class	7
(0.7, 0.8]	Intermediate coordination		8
(0.8, 0.9]	Good coordination		9
(0.9, 1.0]	High quality coordination		10

#### 4.3.3 Influencing factors of coupling coordination degree: Tobit model

Because the coupling coordination value between the two systems of “FM-HED” is always between 0 ~ 1, and as the explanatory variable of the regression model, it can also be called the restricted explanatory variable, so this study uses the Tobit model to estimate to avoid the bias caused by ordinary OLS regression (29). At the same time, in order to avoid the conflict between the fixed effect and the panel Tobit model, the panel Tobit random effect model was selected to analyze the influencing factors of the coupling coordination degree of the “FM-HED” system. The index selection of influencing factors is shown (Table 4).

**Table 4:** Factors influencing the degree of coupling coordination.

Variable name	Symbol definition	Variable description	Mean	Min	Max
Dependent variable	Coupling Coefficient	Calculated result from coupling coordination model	0.470	0.218	0.755
Exploratory variables	R&D Investment Intensity	R&D expenditure/internal expenditure on GDP	0.011	0.003	0.030
	Labor Force Level	Logarithm of the number of employed persons	7.609	5.841	8.853
	Industrial Agglomeration Level	Number of employed persons/administrative area	0.026	0.001	0.217
	Older adult Population Burden	Number of participants in old-age insurance/total population of the region	0.350	0.614	0.031
	Degree of Government Intervention	Fiscal expenditure (general public budgetary expenditure of local governments)/GDP	0.253	0.129	0.627

In general, the most direct and obvious influencing factors in the process of modernization are usually science and technology, and the intensity of R&D investment is selected to measure the impact of science and technology on the degree of coupling coordination (30). The labor factor is the most active existence of various production factors, and the other factors can only operate through labor (31), so the labor force level is selected to reflect the influence of human capital on the coupling coordination degree. The number of employed persons/administrative area is used to express the degree of industrial agglomeration (employment density), which can reflect the employment status and economic development level of a region. In addition, the coupling of FM and HED needs to be guided by the external support of the government (32), that is, the burden of the older adult population and the degree of government intervention, which reflect the implementation of national finance and public policies during the study period, respectively.

In the process of setting up the panel Tobit model, in order to avoid the influence of heteroskedasticity and multicollinearity, this paper takes the logarithmic treatment of the employed persons on the basis of the preliminary test of the correlation characteristics of the variables, and the specific model is constructed as follows:


(6) $$Y_{\mathrm{it}}=\gamma +\delta x_{\mathrm{it}}+\varepsilon$$

Equation 6, $Y_{\mathrm{it}}$ represents the coupling coordination degree of the two systems of “FM-HED” in the *i* region in the*t* period, $x_{\mathrm{it}}$ represents the influencing factors, γ represents the constant term, δ represents the regression coefficient, and ε represents the random disturbance term.

### 4.4 Results and analysis

#### 4.4.1 Temporal analysis of the coupling coordination degree of FM and HED

**Figure 2:**
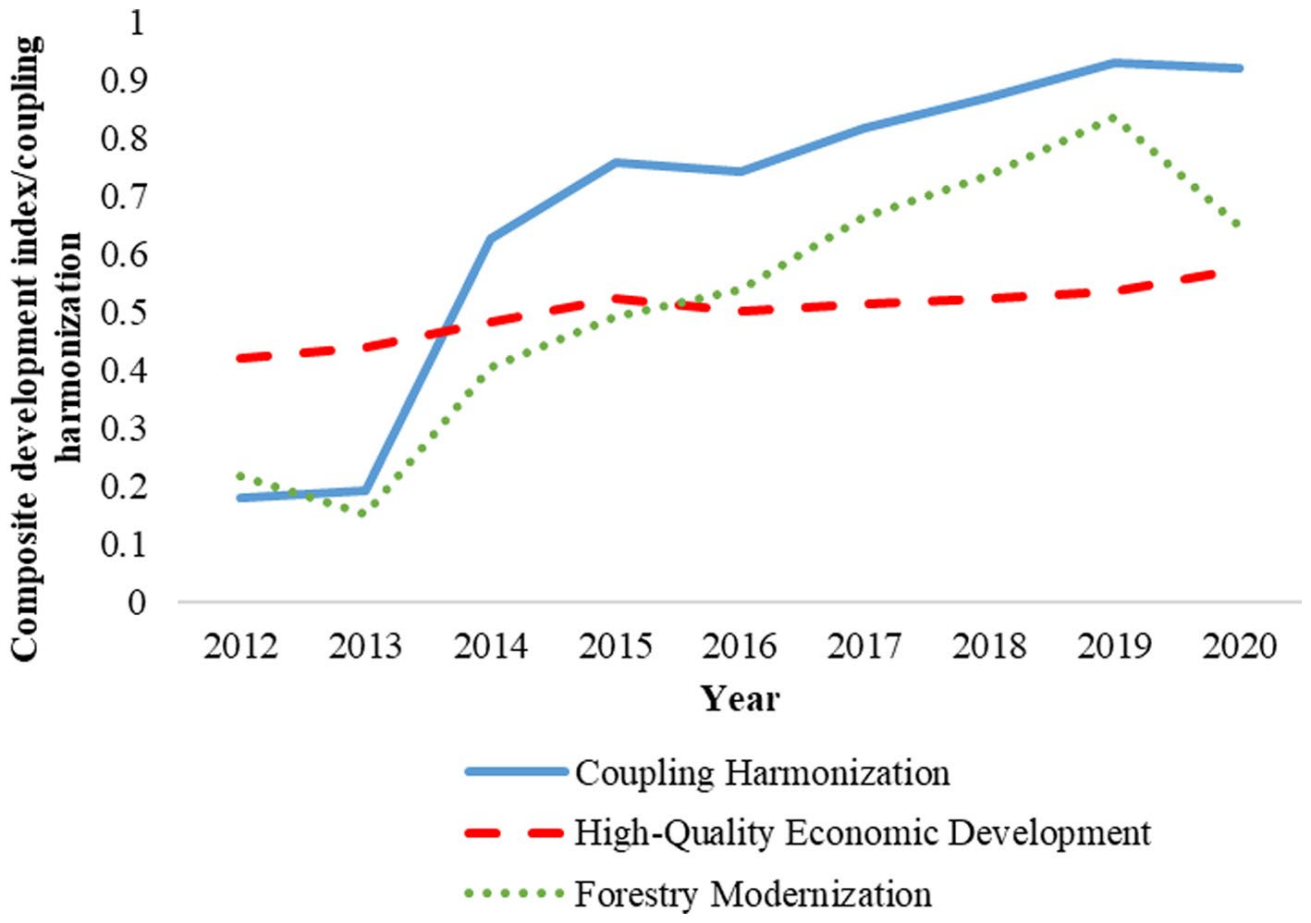
Changes in the time sequence of the coupling coordination of “FM-HED” in China.

Using the entropy value method, combined with the evaluation index system of “FM-HED” coupled and coordinated development constructed in this paper, the 2012 to 2020, the comprehensive development index of China’s FM and HED was further measured by using the coupling coordination degree model to calculate the coupling coordination degree between China’s FM and HED (33), and the time series change analysis of the coupling coordination degree between the comprehensive development index of FM and HED and the coupling coordination degree of the two systems was analyzed. The results of the specific correlation index and coupling coordination degree and their time series changes are shown in Figure 2.

It can be seen from Figure 2 that from 2012 to 2020, the coupling coordination degree of “FM-HED” system showed a dynamic trend of “rapid increase in the early stage and steady improvement in the later stage.” The relationship between the level of HED and the level of FM is divided into 2015, and the level of HED is higher than that of FM before 2015, and the HED gradually lags behind the level of FM after 2015. Specifically, the comprehensive development index of FM system showed an upward trend on the whole, but in 2012 and 2019, the comprehensive development index of FM system decreased significantly. One possible explanation is that, on the one hand, the development of human society must go through three stages: “agriculture, industrialization and informatization.” In 2012, China’s forestry construction entered the green development stage of forestry under the guidance of people’s livelihood (34), which put forward higher requirements for the protection and sustainable management of forest resources. With this, there are higher requirements for the accurate monitoring of basic forestry resource data and the unified planning and construction of forestry land. However, the level of informatization at that time was far from meeting the needs of modern forestry development, and it became a constraint to the development of FM in the short term (35). On the other hand, the outbreak of COVID-19 in 2019. The current epidemic has had varying degrees of impact on afforestation planning (36), afforestation production and management, as well as on people, enterprises, institutions, who rely on forests for their livelihoods. The most severely affected areas are forest tourism, timber trade, and forest financing, ultimately leading to a brief decline in the comprehensive development index of the FM system (37). The comprehensive development index of the HED system has changed relatively slowly, showing an overall upward trend, but slightly decreased around 2015. The main reason may be that during this stage, China’s economic growth rate has declined, investment has slowed down, and foreign trade has declined. The structural contradictions in economic development are still prominent, and there is still a need to deeply promote supply side structural reforms, resulting in short-term economic fluctuations.

It can be seen from the data in Figure 2 combined with the data in Table 2 that the coupling coordination degree of “FM-HED” has been significantly improved, and a good coordinated development situation has been formed in recent years. Specifically, the coupling coordination degree of “FM-HED” was severe dysregulation in 2012 and 2013, and was in the stage of dysfunctional recession. In 2014, it was suddenly promoted to the primary coordination level and was in the stage of coordinated development. Since then, it has stabilized in the stage of coordinated development, and in 2015 and 2016, it was at the intermediate coordination level. In 2017 and 2018, it was in the level of good coordination, and in 2019 and 2020, it was in the level of high-quality coordination.

#### 4.4.2 Spatial differentiation of the coupling coordination degree of FM and HED

**Figure 3:**
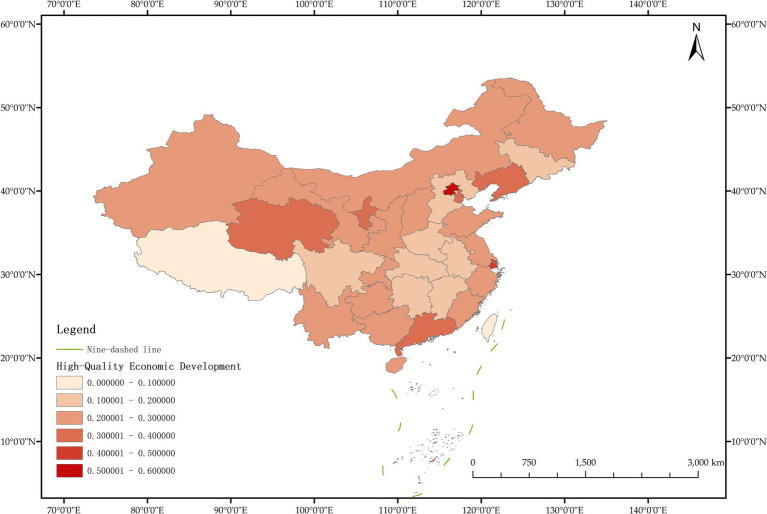
Schematic diagram of HED by provinces and cities in China, 2012.

**Figure 4:**
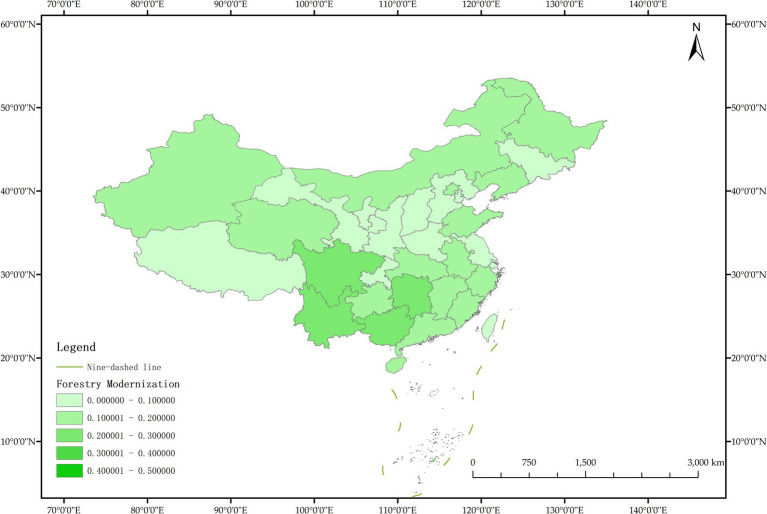
Schematic diagram of FM by provinces and cities in China, 2012.

**Figure 5:**
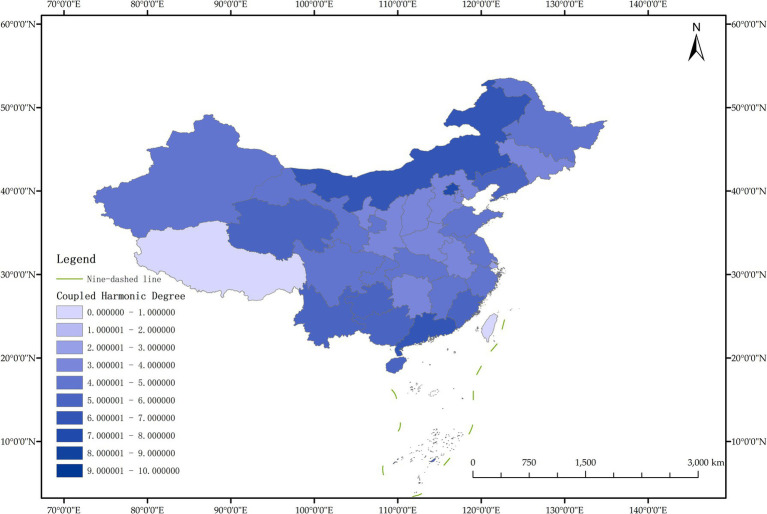
Schematic diagram of the degree of coordination between “FM and HED” by provinces and cities in China, 2012.

**Figure 6:**
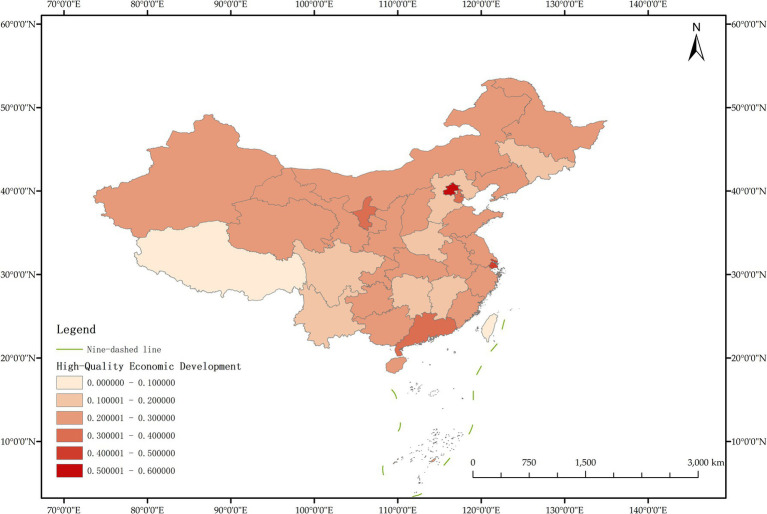
Schematic diagram of HED by provinces and cities in China, 2015.

**Figure 7:**
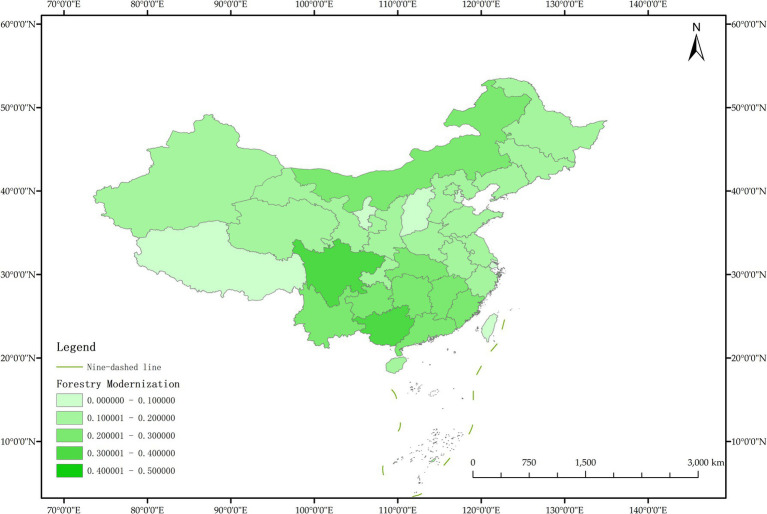
Schematic diagram of FM by provinces and cities in China, 2015.

**Figure 8:**
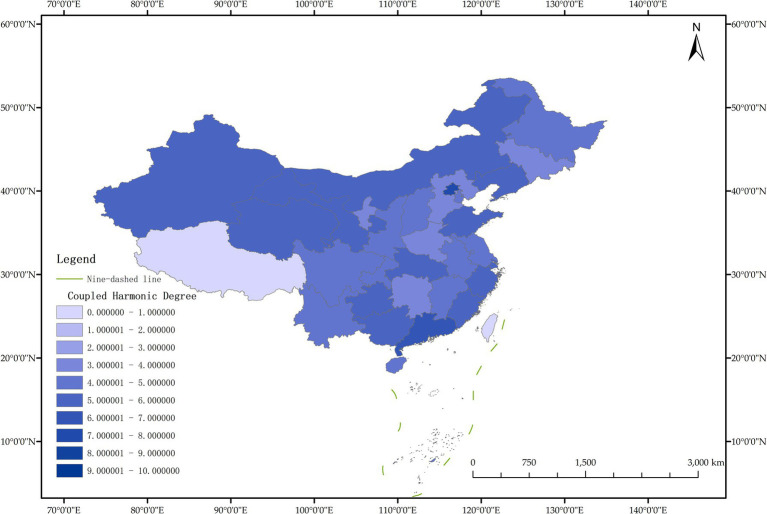
Schematic diagram of the degree of coordination between “FM and HED” by provinces and cities in China, 2015.

**Figure 9:**
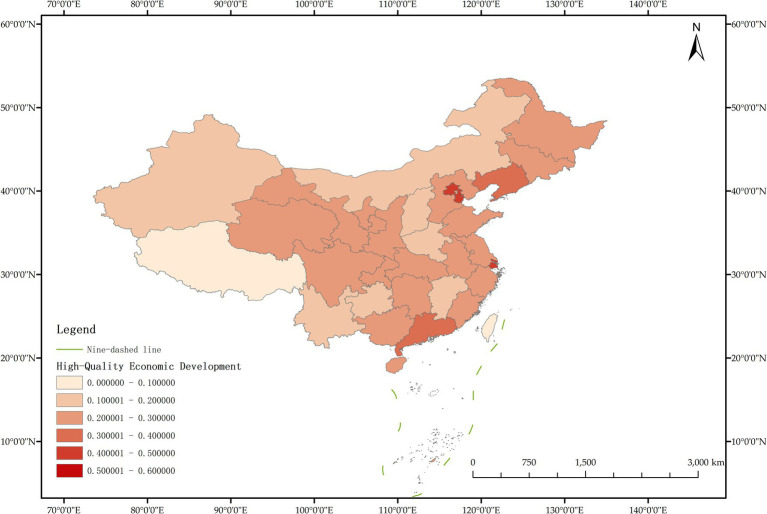
Schematic diagram of HED by provinces and cities in China, 2020.

**Figure 10:**
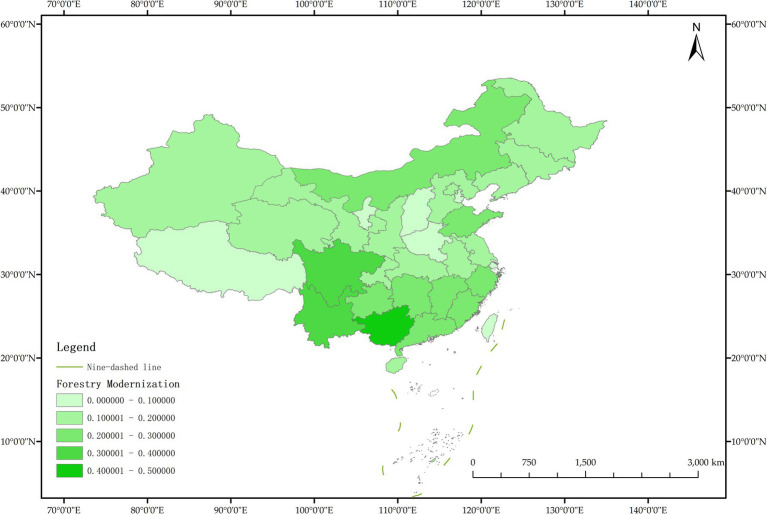
Schematic diagram of FM by provinces and cities in China, 2020.

**Figure 11:**
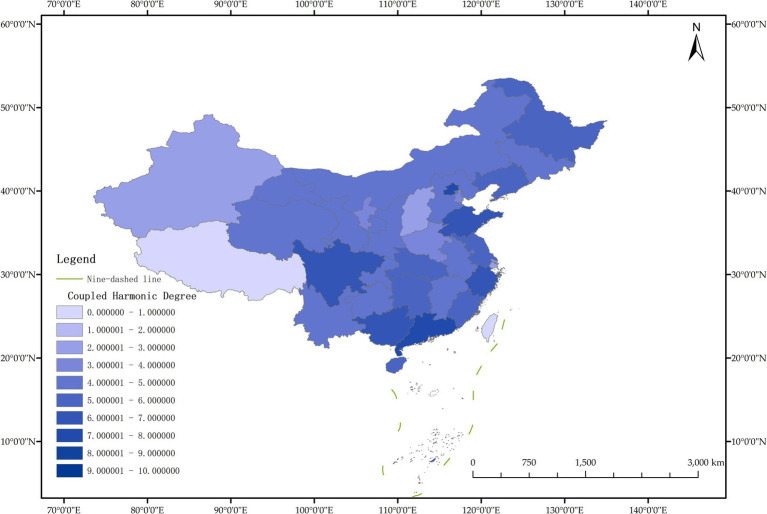
Schematic diagram of the degree of coordination between “FM and HED” by provinces and cities in China, 2020.

The issue of regional disparities in China has always been one of the hot issues that scholars at home and abroad have paid attention to. In order to further analyze the spatiotemporal differentiation characteristics of system coupling, this paper selected three typical years in 2012, 2015 and 2020 to draw the spatial distribution map of FM level, HED level and coupling coordination degree in each region (Figures 3–11).

As can be seen from Figure 5, in 2012, among the 30 provinces and cities in China, the coupling coordination degree of “FM-HED” was in the stage of dysfunctional recession and transitional coordination category, and the coupling coordination degree of each province and city was at a low level as a whole, and only a few provinces and cities were prominent. In terms of spatial distribution, 10 provinces and cities are in the stage of dysfunctional recession, 17 provinces and cities are in the stage of transitional coordination category, and 3 provinces and cities are in the stage of coordinated development class. Beijing is the intermediate level of coordination, and Guangdong and the Inner Mongolia Autonomous Region are the primary level of coordination, both of which belong to the stage of coordinated development. Most of the eastern and western provinces have reached the stage of transitional coordination category. Nine provinces and cities, including Anhui, Hebei, Henan, Hunan, Tianjin, Shanxi, Shaanxi, Jilin and Ningxia Hui Autonomous Region, are mild dysregulation, and they all belong to the stage of dysfunctional recession. In particular, Shanghai has the lowest coupling coordination degree among all provinces and cities, which is moderate dysregulation. As shown in Figures 3, 4, the possible reason is that the level of China’s FM and HED in 2012 was uneven and insufficient.

As can be seen from Figure 8, in 2015, the coupling coordination degree of “FM-HED” in more provinces and cities evolved to the transitional coordination category, and 6 new transitional coordination category provinces and cities were added. Specifically, 5 provinces were mild dysregulation, 11 provinces and cities were on the verge of dysfunction, 12 provinces were barely coordinated, 1 province was initially coordinated, and 1 city was intermediately coordinated. From the perspective of spatial distribution, the coupling coordination degree of “FM-HED” in eastern and western provinces and cities is relatively high. Beijing and Guangdong are still leading in the level of coupling coordination. In 2015, the coupling coordination degree of Shanghai was 0.5291, reaching a state of barely coordination. Combined with Figures 6, 7, it can be seen that compared with the sample base period (2012), FM in most regions of China has made steady progress, the difference between FM and HED has decreased, and the coupling coordination degree has improved. Overall, the level of imbalance between regions has also eased.

Figure 11 shows that in 2020, among the 30 provinces and cities in China, six provinces and cities, including Shandong, Zhejiang, Guangxi Zhuang Autonomous Region, Sichuan, Guangdong, and Beijing, rose to the stage of coordinated development, while the coupling coordination degree of three provinces and cities in Shanxi, Shanghai, and Xinjiang Uygur Autonomous Region declined to varying degrees, and the level of coupling coordination degree of provinces and cities in coastal areas was significantly better than that in inland areas. It can be seen that the coupling and coordinated development of the two systems of “FM-HED” in China still needs to be further run-in, and the differences between inland and coastal areas are worthy of further exploration. In addition, the coupling coordination degree of “FM-HED” in Shanghai has degraded to moderate dysregulation after a short period of improvement, that is, it is the same level as the coupling coordination degree in 2012. The possible reason is that there is a disconnect between the HED of Shanghai and the modernization of forestry, which is ultimately manifested in the decline of the coupling coordination level of “FM-HED” in Shanghai. Combined with Figures 9, 10, Specifically, the comprehensive development index of Shanghai’s HED in 2012, 2015 and 2020 was 0.424, 0.431 and 0.438, respectively, and the comprehensive development index of FM was 0.023, 0.063 and 0.023 fully indicate that Shanghai’s economic level is developing with high quality, but the level of FM fluctuates greatly and remains at a low level for a long time, and the comprehensive index of FM at the end of the observation period even reached only 5.25% of the comprehensive development index of HED. One possible explanation is that compared with other economically developed provinces and cities such as Beijing, Guangdong, and Zhejiang, Shanghai’s green prevention and control coverage rate is low, and it did not reach 10% of the planned target for two consecutive years in 2019 and 2020. At the same time, the contribution of Shanghai’s forestry economy to the overall GDP is too small, which may cause a lack of enthusiasm of local governments, especially in Shanghai, which is centered on finance, and the promotion of FM is easy to fall into the form and ignore the utility, and it is difficult to realize the effective transformation of ecological benefits into economic benefits.

Overall, from 2012 to 2020, the coupling coordination degree of “FM-HED” in 30 provinces and cities in China was not ideal, and the overall development quality was not high. The data analysis results show that the forestry construction of most provinces and cities has not yet been modernized, the quality of economic development still needs to be improved, the two independent systems have not achieved effective mutual promotion, and therefore have not shown the overall effect of “1 + 1 > 2,” and the coordinated development of FM and HED has a long way to go.

#### 4.4.3 Analysis of the influencing factors of the coupling coordination degree of FM and HED

From the above analysis, it can be seen that there are obvious spatial differences in the coupling coordination degree of China’s “FM-HED” system. In order to further investigate the heterogeneity between regions, this study not only performed panel Tobit regression on the coupling coordination degree across the country, but also performed panel Tobit regression on the four major regions (East China, Northeast China, Central China, and Northwest China) to further analyze the influencing factors of the coupling coordination degree between FM and HED, and the specific results are shown in Table 5.

**Table 5:** Benchmark regression results of the influencing factors of coupling coordination degree between FM and HED.

Index/region	National level	Eastern	Northeastern	Central	Northwestern
R&D Investment Intensity	4.044^***^	−0.832	−8.039^**^	22.921^***^	4.930
	(2.640)	(−0.600)	(−2.290)	(5.580)	(0.840)
Labor Force Level	0.018	0.134^***^	0.329^***^	−0.021	0.073^**^
	(0.690)	(2.870)	(10.110)	(−0.260)	(2.490)
Industrial Agglomeration Level	−0.016	0.446	6.654^***^	−5.329	−1.891
	(−0.040)	(0.760)	(3.260)	(−1.510)	(−0.510)
Older Adult Population Burden	−0.548^**^	−0.061	−0.732^**^	0.833	−0.541
	(−2.080)	(−0.180)	(−2.240)	(1.630)	(−0.970)
Degree of Government Intervention	0.139	1.062^***^	0.771^***^	−0.117	0.122
	(1.060)	(4.970)	(5.470)	(−0.260)	(0.580)
cons	0.354	−0.718^*^	−2.050^***^	0.280	−0.041
	(1.620)	(−1.800)	(−7.890)	(0.390)	(−0.150)
log(Sigma)	0.097^***^	0.133^***^	0.020^***^	0.044^***^	0.054^***^
	(7.270)	(4.090)	(7.350)	(9.530)	(12.210)
Likelihood ratio test	*x*^2^ = 301.62 *p* = 0.000	*x*^2^ = 170.83 *p* = 0.000	*x*^2^ = 321.250 *p* = 0.000	*x*^2^ = 39.510 *p* = 0.000	*x*^2^ = 13.190 *p* = 0.000

* means *p* < 0.1, ** means *p* < 0.05, *** means *p* < 0.01, and the *z*-value in parentheses is the same below. The eastern region includes: Beijing, Tianjin, Shanghai, Hebei Province, Shandong Province, Jiangsu Province, Zhejiang Province, Fujian Province, Guangdong Province, Hainan Province; the central region includes: Shanxi Province, Henan Province, Hubei Province, Anhui Province, Hunan Province, Jiangxi Province; the western region includes: Inner Mongolia Autonomous Region, Xinjiang Uygur Autonomous Region, Ningxia Hui Autonomous Region, Shaanxi Province, Gansu Province, Qinghai Province, Chongqing, Sichuan Province, Guangxi Zhuang Autonomous Region, Guizhou Province, Yunnan Province; the northeastern region includes: Heilongjiang Province, Jilin Province, Liaoning Province. (Due to data limitations, this does not include the Xizang, Hong Kong, Macao, Tibet and Taiwan regions).

From the national level, the intensity of R&D investment is significant at the 1% significance level, the burden of the older adult population is significant at the 5% significance level, and the labor force level, industrial agglomeration degree and government intervention degree do not pass the significance test. Among them, the intensity coefficient of R&D investment is positive, indicating that the larger the proportion of R&D expenditure in GDP, the more conducive to the coupling and coordinated development of the two systems. On the one hand, science and technology, as an important symbol of advanced productive forces, are the strategic support for modernization, and on the other hand, science and technology can optimize the efficiency of resource allocation and improve the level of total factor productivity, thereby promoting HED. The aging of the population will increase the burden on the government, and this paper uses the proportion of the number of people insured by pension insurance in the total number of people in the region to approximate the effect of the burden of the older adult, and the coefficient of the burden of the older adult population is negative, indicating that the more serious the aging of the population, the more unfavorable it is to the coupling and coordinated development of the two systems. This is due to the fact that the older adult population is not only unable to participate in forestry production and economic construction as an effective labor force, but also needs to occupy social resources to support them, which to a certain extent squeezes out the resources required for FM and HED, and passively becomes an obstacle in the coupling process of the two systems.

From the perspective of the four regions, the specific analysis is as follows:

First, the regression coefficient of R&D investment intensity is significantly negative in Northeast China and positive in Central China. One possible explanation for this is that compared with the relatively developed infrastructure conditions in central China, the process of transforming forestry scientific and technological achievements into productivity in Northeast China is relatively slow, and although Northeast China has accumulated a lot of R&D investment, these research results have not been transformed into practical applications and productivity in a timely and effective manner. In addition, due to the influence of historical and geographical factors, there may be greater pressure on economic transformation, and the problem of shrinking market or insufficient demand will lead to poor R&D investment, which will affect the coupling relationship between FM and HED. The market demand in the central region is stronger, and enterprises are more willing to apply R&D results, which also promotes the effective synergy between FM and HED. From 2012 to 2020, the relative level of innovation output and transformation in Northeast China was 5.19–7.03, far lower than that in the Beijing-Tianjin-Hebei region (15.88–21.04) and the Yangtze River Delta region (11.12–13.01), and there was also a certain gap with the national average, and this gap still has a tendency to further expand over time (38).

Second, the regression coefficient of labor force level in East China, Northeast China and Northwest China is significantly positive, which indicates that the accumulation of human capital is a prerequisite for the realization of FM in most provinces and cities in China, and can effectively improve the quality of economic development. The only thing that is not significant is in central China. Central China is located in the interior of the country, and to a certain extent, it can enjoy the economic radiation of the eastern region, which is conducive to FM and HED (39). But at the same time, Central China will also be affected by the “siphon phenomenon” in the surrounding areas, resulting in brain drain (40). This hinders FM and HED, and ultimately leads to the separation of FM and HED and human capital accumulation in Central China (41).

Third, the regression coefficient of industrial agglomeration in Northeast China is significantly positive, which fully shows that FM and HED in Northeast China depend on industrial agglomeration, because Northeast China is dominated by state-owned forests, public welfare forests occupy the vast majority, and the development is strictly restricted.

Fourth, the regression coefficient of the burden of the older adult population in Northeast China is significantly negative, indicating that the burden of the older adult population in Northeast China has been relatively serious, which has affected FM and HED. From a social perspective, the economic development of Northeast China has been weak in recent years, and a large number of young and middle-aged laborers have outflowed to other regions. From the perspective of forest areas, the low income of forest workers and the poor quality of life in forest areas also make young and middle-aged laborers in forest areas choose to leave forestry, which further aggravates the problem of population aging in Northeast China.

Fifth, the regression coefficient of the degree of government intervention in East China and Northeast China was significantly positive. The possible explanation is that in East China, where high-tech industries are the mainstay, and in Northeast China, where heavy industry is dominant, most of the infrastructure investment necessary for forestry modernization is borne by the government. Modern forestry is a capital-intensive industry, and the capital investment in the process of forestry modernization development mainly comes from the government’s transfer payment, and the external environment mainly depends on the support of policies such as forest product price protection, tax incentives and import and export.

In order to further test the robustness of the results, the Tobit panel regression was performed as a robustness test by leaving the explanatory variables unchanged and removing the non-significant explanatory variables, as shown in Table 6. From the national level, the intensity of R&D investment and the burden of the older adult population are both significant at the level of 1%, and the magnitude and direction of the regression coefficient have not changed significantly, which is consistent with the benchmark regression results of the influencing factors of the coupling coordination degree between FM and HED. From the perspective of the four regions, the significance of East China and Northwest China remained almost unchanged, the robustness test results and benchmark regression results of Northeast China were of high consistency, and the significance of R&D investment intensity variables in Central China also did not change. In general, the benchmark regression of the influencing factors of coupling coordination between FM and HED has good robustness.

**Table 6:** Robustness test results of the influencing factors of coupling coordination degree between FM and HED.

Index/region	National level	Eastern	Northeastern	Central	Northwestern
R&D Investment Intensity	3.914^***^	–	−8.039^**^	21.381^***^	–
	(2.690)	–	(−2.290)	(5.380)	–
Labor Force Level	–	0.317^***^	0.329^***^	–	0.041^**^
	–	(4.250)	(10.110)	–	(2.550)
Industrial Agglomeration Level	–	–	6.654^***^	–	–
	–	–	(3.260)	–	–
Older Adult Population Burden	−0.646^***^	–	−0.732^**^	–	–
	(−2.740)	–	(−2.240)	–	–
Degree of Government Intervention	–	0.970^***^	0.771^***^	–	–
	–	(7.280)	(5.470)	–	–
cons	0.545^***^	−2.132^***^	−2.050^***^	0.155^***^	0.161
	(12.06)	(−3.730)	(−7.890)	(3.120)	(1.370)
log(Sigma)	0.099^***^	0.025^***^	0.020^***^	0.047^***^	0.038^***^
	(7.370)	(6.070)	(7.350)	(3.140)	(3.780)
Likelihood ratio test	*x*^2^ = 303.330	*x*^2^ = 58.846	*x*^2^ = 321.250	*x*^2^ = 25.210	*x*^2^ = 19.970
	*p* = 0.000	*p* = 0.000	*p* = 0.000	*p* = 0.000	*p* = 0.000

* means *p* < 0.1, ** means *p* < 0.05, *** means *p* < 0.01, and the *z*-value in parentheses is the same below.

## 5 Conclusion and policy suggestions

### 5.1 Conclusion

Based on the panel data of 30 provinces and cities (excluding Xizang, Hong Kong, Macao and Taiwan) from 2012 to 2020, this paper constructs an evaluation index system for the coupling and coordinated development of “FM-HED,” and measures the coupling coordination degree of the “FM-HED” system in each province, and the temporal and spatial differentiation of the coupling coordination system of FM and HED in each region were analyzed, and the factors affecting the coupling coordination degree of FM and HED were found, and the conclusions are as follows.

1. The results of the time series analysis of the coupling coordination degree of “FM-HED” show that the coupling coordination degree of FM and HED system from 2012 to 2020 shows a dynamic trend of “rapid increase in the early stage and steady improvement in the later stage.” The comprehensive development index of FM and HED was alternated in 2015, and the comprehensive development index of HED before 2015 was higher than that of FM, and the comprehensive development index of HED after 2015 was lower than that of FM. During this period, the coupling coordination degree of “FM-HED” has been significantly improved, and it has experienced five levels: severe dysregulation, primary coordination, intermediate coordination, good coordination, high-quality coordination, and has formed a good coordinated development situation in recent years.
2. The results of the spatial differentiation of the coupling coordination degree of “FM-HED” show that the coupling coordination degree of “FM-HED” in 30 provinces and cities in China from 2012 to 2020 is not ideal, and the overall development is uneven. Specifically, in 2012, the level of FM and HED in China showed obvious imbalance and inadequacy, and the coupling coordination degree of most provinces and cities was at a low level, and the distribution of a few provinces and cities with a high coupling coordination degree was extremely scattered. In 2015, the coupling coordination degree between the eastern and western provinces and cities improved, the gap between the level of FM and the level of HED in most regions of China narrowed, and the imbalance between regions was also alleviated. In 2020, as the coupling coordination degree of six provinces and cities including Beijing rose to the stage of coordinated development, the difference in the level of coupling coordination between provinces and cities in coastal areas and provinces and cities in inland areas became increasingly obvious, and coastal areas were significantly better than inland areas.
3. The results of the analysis of the influencing factors of the coupling coordination degree of “FM-HED” show that from the national level, the intensity of R&D investment has a significant positive effect on the coupling and coordinated development of the two systems at the 1% significance level, and the burden of the older adult population has a significant negative promotion effect on the coupling and coordinated development of the two systems at the 5% significance level, and the labor force level, the degree of industrial agglomeration and the degree of government intervention do not pass the significance test. From the perspective of the four regions, each index only has a significant impact on the system coupling and coordinated development in a specific region, the role of R&D investment intensity is reflected in Northeast China and Central China, the labor force level has a significant positive effect on the three regions outside Central China, the degree of industrial agglomeration only affects the coupling and coordinated development of the two systems in Northeast China, the burden of the older adult population has a significant negative impact in Northeast China, and the role of government intervention is mainly reflected in East China and Northeast China.

### 5.2 Policy suggestions

The data analysis results show that from 2012 to 2020, the development level of coupling coordination degree of “FM-HED” in China has increased year by year, but the development level of coupling coordination degree between the two systems has not yet made breakthrough progress in some provinces and cities, which leads to obvious spatial heterogeneity. In order to promote the coupling coordination of the two systems, the following policy suggestions are put forward.

(1) Pay attention to the strong and weak changes in the level of FM and HED, and make up for the shortcomings in time to ensure that the two systems are in a positive state of mutual stimulation, so as to achieve coordinated development.

When forestry development lags behind economic development, there is a need for more in-depth exploration of the infrastructure and technology aspects related to forestry production.

First, it is necessary to realize forestry mechanization and forestry informatization, and constantly arm the forestry development process with modern material conditions, so as to improve the total factor productivity of forestry and promote the coordination and synchronization of FM and economic development.

Second, attention should be paid to improving the construction of forestry infrastructure, opening up the key channels for the exchange of products between forest areas and the outside world, and promoting the exchange of materials inside and outside the forest area and the forestry industry, so as to make modern forestry an important driving force for HED.

When economic development lags behind forestry development, it is necessary to promote the green production of enterprises and build a green economic development system.

First, the government should give high support to the realization of the value of low-carbon economy, green finance and forest products, and provide tax incentives, credit incentives, financial subsidies and other support policies for the green development of relevant enterprises.

Second, in the context of the policy goal of “carbon peak and carbon neutrality,” the concept of green development should be used to guide the supply-side structural reform, and gradually improve the green development level of the national and provincial economies.

(2) Establish cross-regional forestry development planning and economic cooperation, improve the utilization efficiency of various resources, and avoid the constraints of resource mismatch and the coupling system of “FM - HED.”

In order to promote the coordinated development of provinces and cities, it is necessary to continue to formulate forestry development and economic development strategies from the overall situation.

First, vigorously promote the network sharing of preferential policies, human capital and other resources, and explore diversified cooperation mechanisms.

Second, rely on the core region to drive the forestry construction of neighboring regions to form a regional economic network with win-win cooperation; strengthen the support of coastal areas to inland areas, so as to narrow the development gap between provinces and cities.

Third, for the eastern region, more resources should be invested to promote FM on the basis of maintaining and improving the quality of economic development, and for the central and western regions, we should make full use of the gradient advantages of economic development, make up for the development gap with the help of eastern resources, and strive to improve the level of HED.

(3) Adhere to the principle of adapting measures to local conditions, and implement differentiated management according to the development of the coupling coordination system of FM and HED in various provinces and cities. For example, Central China should make full use of the more developed infrastructure, increase the intensity of R&D investment, and promote the accelerated transformation of forestry scientific and technological achievements. East China, Northeast China and Northwest China should give full play to the leading role of human capital in economic development, use better living conditions to “attract and retain people,” alleviate the current situation of aging, and achieve both the quantity and quality of the labor force. In addition, the forest resources in Northeast China are dense and contiguous, and the coordinated development of HED and FM is more dependent on industrial agglomeration, so it is necessary to establish forestry industrial parks in a timely manner to assist in incubating forestry innovation and entrepreneurship projects, so as to extend the industrial chain and improve the value realization process of forest products. The degree of government intervention in East China and Northeast China is relatively high, and fiscal transfer payments and preferential support policies have become an important support in the development process of the coupling coordination system of FM and HED.

## Data Availability

The datasets presented in this article are not readily available because the privacy and confidentiality security of the laboratory. Requests to access the datasets should be directed to SW, wanshenwei@ruc.edu.cn.

## Footnotes

^4^Guidelines for the Development of the National Forest Economy (2021–2030). https://www.gov.cn/zhengce/zhengceku/2021-12/04/content_5655829.htm.

^5^The First Proposal and Development of “China’s Forestry Modernization” https://news.sciencenet.cn/htmlnews/2017/6/380037.shtm.
